# Evaluation of suitable reference genes for normalization of quantitative reverse transcription PCR analyses in *Clavibacter michiganensis*

**DOI:** 10.1002/mbo3.928

**Published:** 2019-10-02

**Authors:** Na Jiang, Qingyang Lyu, Sining Han, Xin Xu, Ronald R. Walcott, Jianqiang Li, Laixin Luo

**Affiliations:** ^1^ Department of Plant Pathology, College of Plant Protection China Agricultural University Beijing China; ^2^ Department of Plant Pathology, 4315 Miller Plant Sciences the University of Georgia Athens GA USA

**Keywords:** *Clavibacter michiganensis*, geNorm, NormFinder, real‐time qRT‐PCR, reference genes

## Abstract

*Clavibacter michiganensis*, the causal agent of bacterial canker of tomato, is a Gram‐positive bacterium and a model for studying plant diseases. The real‐time quantitative reverse transcription PCR (real‐time qRT‐PCR) assay is widely used to quantify gene expression in plant pathogenic bacteria. However, accurate quantification of gene expression requires stably expressed reference genes that are consistently expressed during the experimental conditions of interest. The use of inappropriate reference genes leads to a misinterpretation of gene expression data and false conclusions. In current study, we empirically assessed the expression stability of six housekeeping genes (*gyrB*, *rpoB*, *tufA*, *bipA*, *gapA,* and *pbpA*) of *C*. *michiganensis* under five experimental conditions using two algorithms, geNorm and NormFinder. *C*. *michiganensis* expressed *gyrB*, *bipA,* and *gapA* stably when growing in nutrient‐rich broth (TBY broth and modified M9 broth). We concluded that *pbpA*, *tufA,* and *gyrB* were suitable reference genes in *C*. *michiganensis*—tomato interaction studies. We also recommended *bipA* and *rpoB* to be used to study bacterial gene expression under nutrient‐poor conditions. Finally, *gyrB*, *pbpA,* and *rpoB* can be used to normalize the quantification of *C*. *michiganensis* gene expression while the bacterium is in the viable but nonculturable (VBNC) state. This study identified the most suitable reference genes depending on the experimental conditions for calibrating real‐time qRT‐PCR analyses of *C*. *michiganensis* and will be useful in studies that seek to understand the molecular interactions between *C*. *michiganensis* and tomato.

## Graphical abstract

We evaluated the most suitable reference genes for qPCR analyses in *Clavibacter michiganensis*, the causal agent of bacterial canker in tomato. We recommend *bipA* and *rpoB* to be used to study bacterial gene expression under nutrient poor conditions, and *pbpA*, *tufA* and *gyrB* are suitable reference genes in *C. michiganensis* ‐ tomato interaction studies. Finally, *gyrB*, *pbpA* and *rpoB* can be used to normalize the quantification of *C. michiganensis* gene expression while the bacterium is in the viable but non‐culturable (VBNC) state.

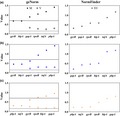

## 1 INTRODUCTION

Species within *Clavibacter* genus are xylem colonizing Gram‐positive bacteria. Based on average nucleotide identity from whole genome analyses, *Clavibacter michiganensis* subspecies have been to elevate to the species level recently (Li et al., 2018; Tambong, 2017). Specific *Clavibacter* species can infect different hosts, and *Clavibacter michiganensis* (also known as *C.* *michiganensis* subsp. *michiganensis*) is the causal agent of bacterial canker of tomato (*Solanum lycopersicum*) (Davis, Gillaspie, Vidaver, & Harris, 1984; Strider, 1969). This bacterium has become a model for studying the mechanisms of pathogenicity of Gram‐positive bacteria due to its potential risk of causing substantial economic losses in tomato production and the availability of its whole genome sequences (Gartemann et al., 2008; Thapa et al., 2017). Relative to most economically important phytopathogenic bacteria that are in the Proteobacteria phylum, less attention has been paid to *C*. *michiganensis* with regards to molecular host‐pathogen interactions (Mansfield et al., 2012). The mechanisms of pathogenicity for *C*. *michiganensis* are substantially different to those Proteobacteria members (Eichenlaub & Gartemann, 2011). The genome of *C*. *michiganensis* strain NCPPB382 was published in 2007 and provides a valuable resource for exploring its pathogenesis (Gartemann et al., 2008). Studies of the molecular biology of bacterial canker of tomato can ultimately improve disease management. The survival and transmission of *C*. *michiganensis* on/in the seed or in the field is important for understanding and managing tomato bacterial canker (Fatmi & Schaad, 2002). Specifically, *C*. *michiganensis* cells on tomato seeds may be induced into the viable but nonculturable (VBNC) state by a range of conditions including bactericide (copper agent) treatments, dry conditions, and lack of available nutrients and may pose a risk for tomato production (Jiang et al., 2016; de León, Siverio, López, & Rodríguez, 2011). The VBNC state of some Gram‐negative phytopathogenic bacteria has been shown to be a survival strategy in response to harsh environmental conditions (Oliver, 2010). However, there are very few studies of the molecular mechanisms involved in the induction and maintenance of VBNC state of phytopathogenic bacteria.

Real‐time quantitative reverse transcription polymerase chain reaction (real‐time qRT‐PCR) has become a powerful technique for RNA quantifying and the expression of specific genes (Die & Román, 2012). This assay was used to study the interactions between *C*. *michiganensis*—tomato plants, and the results revealed that the difference of gene expression between systemic and located infection of *C. michiganensis*, while some genes located on the pathogenicity island of *C. michiganensis* were involved in the suppression of tomato basal defenses (Chalupowicz et al., 2017, 2010). qRT‐PCR was also used to validate the expression patterns obtained by cDNA‐amplified fragment length polymorphism (AFLP) and identified five candidate genes for developing *C*. *michiganensis*—tolerant tomato cultivars (Lara‐Ávila, Isordia‐Jasso, Castillo‐Collazo, Simpson, & Alpuche‐Solís, 2011). Moreover, real‐time qRT‐PCR was used to detect VBNC cells of bacteria, because sustainable gene expression is a reliable indicator for cell viability (Oliver, 2010; Liu, Wang, Tyrrell & Li, 2010). For example, the expression of housekeeping genes, such as 16S rRNA and *rpoS* (RNA polymerase and sigma factor), was quantified to reveal the variation in viability of bacterial cells (González‐Escalona, Fey, Höfle, Espejo, & A Guzmán, 2006).

Obtaining accurate gene expression results by real‐time qRT‐PCR is difficult in some cases, due to the shortage of stably expressed reference genes for data normalization and quantification. The expression of prokaryotic housekeeping gene, like 16 rRNA, varied widely under different conditions (Takle, Toth, & Brurberg, 2007; Vandecasteele, Peetermans, Merckx, & Eldere, 2001). Some software or algorithms, such as geNorm, NormFinder, BestKeeper, and ∆*C*_t_, have been developed to evaluate the expression stability of housekeeping genes in different experiments (Andersen, Jensen, & Ørntoft, 2004; Pfaffl, Tichopad, Prgomet, & Neuvians, 2004; Silver, Best, Jiang, & Thein, 2006; Vandesompele et al., 2002).

Despite the comments above, the empirical validation of *C*. *michiganensis* reference genes for use in real‐time qRT‐PCR analyses received few attention. The expression stability of 3 housekeeping genes, *gyrA*, *bipA*, and *qcrA*, was estimated by geNorm under experimental conditions and as a result, *gyrA* and *bipA* were used as reference genes in the study of interactions between tomato and *C*. *michiganensis* (Chalupowicz et al., 2017, 2010). Since only three housekeeping genes were evaluated under one experimental condition, it is unlikely that these genes will be suitable for normalizing gene expression studies conducted in other experiments.

In current study, the expression stabilities of six housekeeping genes from *C*. *michiganensis* were tested under five different conditions. The results provide information on the most suitable reference genes for gene expression studies in *C*. *michiganensis* and other phytopathogenic bacteria.

## 2 MATERIALS AND METHODS

### 2.1 Bacterial strains and culture conditions

*Clavibacter michiganensis* strain BT0505 (isolated from tomato field in Inner Mongolia autonomous region of China in 2005) was grown in TBY broth (5 g/L yeast extract, 5 g/L NaCl, and 10 g/L tryptone) at 28°C with shaking (140 rpm) for 22 hr (to an OD_580nm_ of *ca.* 1.0). The cells were harvested by centrifugation at 14,000 *g* for 3 min and washed three times with a 0.85% (w/v) NaCl solution before treatment.

Five experimental conditions were used for bacterial RNA extraction and candidate genes expression. TBY broth was used as a standard medium, modified M9Cmm‐minimal (mM9) medium (11.28 g/L 5 × M9 minimal salts, 2 mM MgSO_4_·7H_2_O, 0.01 mM CaCl_2_·2H_2_O, 0.5 mg/L Vitamin B1, 0.5 mg/L nicotinic acid, and 3.96 g/L glucose) was a basic medium with less nutrients, and tomato seedling homogenate (TSH) medium was used to mimic the natural host environment and the final concentration of TSH was adjusted to 10% (Flügel, Becker, Gartemann, & Eichenlaub, 2012). *Clavibacter michiganensis* cells at late log phase were added to TBY broth, mM9 medium, and TSH medium at the ratio of 1:100, respectively, and the final titer of bacteria was *ca.* 10^7^ CFU/ml. The growth curve of *C*. *michiganensis* in these three media was measured with a Bioscreen C Pro Automated Microbiology Growth Curve Analysis System (Oy Growth Curve Ab Ltd.) at 28°C with continuous shaking and calculated by Prism 6 (GraphPad Software). Time points representing log, stationary, and decline phases were selected for RNA extraction according to the growth curves.

The other two experimental conditions were 0.85% NaCl solution supplemented with and without 50 μM of CuSO_4_. These conditions represent a VBNC induction condition and a starvation treatment, respectively. *Clavibacter michiganensis* cells at late log phase were added into 0.85% NaCl solution with and without 50 μM CuSO_4_ to an OD_580nm_ of 0.45 (*ca.* 10^8^ CFU/ml), respectively. Cells were incubated at 28ºC without shaking and harvested samples at 12, 24, and 48 hr, respectively. The culturable cells in the starvation treatment were assessed by spreading serial dilutions on TBY agar incubating at 28°C for 3 days and counting the colonies. In contrast, the VBNC cells were counted as described previously reported method (Jiang et al., 2016). This experiment included two biological replicates of each treatment, and each biological replicate included three technical replicates in survival curves analysis. During real‐time qRT‐PCR analyses, each treatment had three biological replicates.

### 2.2 RNA isolation and cDNA synthesis

To extract RNA, 1 ml of *C*. *michiganensis* cells (OD_580nm_ = 1.0) was harvested by centrifugation at 14,000 *g* for 5 min at 4°C at each time point in different treatment and stored at −80°C immediately. Total RNA was extracted using the SV Total RNA Isolation System (Promega Corporation) according to the manufacturer's instructions with minor modifications. Cells were resuspended in 100 μl freshly prepared 1 × TE buffer containing 50 mg/ml lysozyme and incubated at 37°C for 10 min, then the manufacturer's protocol was followed. RNA was eluted in 70 μl of nuclease‐free water and kept at −80°C. Total RNA concentration and purity were determined using a NanoDrop 2000 (Thermo Fisher Scientific), and RNA integrity was tested by running 1% (w/v) native agarose gel in 1 × TAE buffer under 6 V/cm for 15 min. Two bands will be observed on the gel after EB staining, the intensity of 23S rRNA band should be twice as strong as the 16S rRNA band if the integrity of RNA sample is good. The concentration of all RNA samples was adjusted to 100 ng/μl and used for following experiment.

Reverse transcription was performed on 2 μl (200 ng) of RNA with the PrimeScript RT Reagent Kit with gDNA Eraser (Takara, Kusatsu, Shiga, Japan). The cDNA was fourfold diluted with EASY Dilution Solution for Real‐Time PCR (Takara, Kusatsu, Shiga, Japan) and stored at −20°C. All cDNA samples were tested by conventional PCR using the primer pair rRNA‐I (5′‐CATCACAGCCAGCCATC‐3′)/rRNA‐O (5′‐ACCACCGTTATCCAGGC‐3′) to confirm the absence of genomic DNA (gDNA). The forward primer, rRNA‐I was located between positions 2,253,240 and 2,253,256 and the reverse primer, rRNA‐O was located between positions 2,253,739 and 2,253,755 according to the genome sequence of *C*. *michiganensis* strain NCPPB382 (GenBank: AM711867.1) (Gartemann et al., 2008).

### 2.3 Oligonucleotide primer design

The DNAMAN 6 software (Lynnon Biosoft) was used to design primers based on the genome sequence of *C*. *michiganensis* strain NCPPB382 to amplify the corresponding sequences of candidate *genes* from strain BT0505. Oligonucleotide primers were synthesized by Sangon Biotech (Shanghai, China), and the amplicons were sequenced by Life technologies (Beijing, China). Based on the sequence data, eight pairs of primers were selected for real‐time qRT‐PCR and used for gene expression analyses (Table 1). The amplicon size and the annealing temperature ranged from 50 to 210 bp and 55 to 60°C, respectively. The specificity of each primer pair was determined by SYBR Green‐based real‐time PCR followed by electrophoresis in 2% (w/v) agarose gel and melt curve analyses. The amplification efficiency of each primer pair was obtained from the cDNA dilution ratio—*C*_t_ value standard curve. The curve was generated with fourfold diluted cDNA template and the slope of the curve was used in the efficiency equation: E = (10^1/slope^‐1)*100. The primer pair was chosen when the efficiency was between 90% and 110% and the correlation index *R* ^2^ was >0.99.

**Table 1:** *Clavibacter michiganensis* genes and oligonucleotide primers used for real‐time qRT‐RCR analyses

Gene name	Gene description	GenBank accession number	Primer sequence	Amplicon size (bp)	E^a^ (%)
*gyrB*	DNA gyrase B subunit	KJ123741	RT‐F: GGACAGCACATCACGACCC	202	91
			RT‐R: CCTTCGGCATCTTCTTCCC		
*pbpA*	Penicillin‐binding protein 2	KJ123735	RT‐F: CGACGCCACTGATTCTCG	105	94
			RT‐R: GCTTCGCCGACCCGAT		
*gapA*	Glyceraldehyde−3‐phosphate dehydrogenase	KJ123733	RT‐F: TTGACCTGGTTGCCGATGAC	64	101
			RT‐R: TCAACGACCCGCACTCCTC		
*bipA*	Predicted membrane GTPase involved in stress response	KF990998	RT‐F: GGGTGCTGGTCGTCGTA	63	93
			RT‐R: CGAGCCGCTGTTCAAG		
*tufA*	Translation elongation factor Tu	KJ123740	RT‐F: CAGGAGCCCGCAGTTCT	89	101
			RT‐R: GTCCCACCGTCAAGACC		
*rpoB*	DNA‐directed RNA polymerase beta subunit	KJ123738	RT‐F: ATGCCGAGCGGATTGAG	115	101
			RT‐R: GCAACAAGGGCGTCATCT		
*chpC*	Predicted serine protease	KJ123731	RT‐F: CCATCGTGCTCTGCTCTGT	148	91
			RT‐R: CGAAGCGGAGACCAAGC		
*celA*	Endo−1,4‐beta‐glucanase	KJ123730	RT‐F: CATTTCGCCAAGTTCGTG	55	98
			RT‐R: AACGCAAGGAGAAGGACG		

a Amplification efficiency, calculated as E = (10^1/slope^−1)*100, where the slope was obtained from the cDNA dilution ratio – *C*_t_ value standard curve.

### 2.4 Conventional PCR and qPCR analyses

The primers listed in Table A1 in Appendix were used to amplify the six candidate reference genes and two validating genes. *Clavibacter* *michiganensis* cells suspended in sterile water at a final titer of *ca.* 10^8^ CFU/ml were used for the PCR assays. The 25 µl reaction mixtures for conventional PCR contained 1 × *Taq* reaction buffer, 240 μM dNTPs, 0.4 μM each primer, 0.05 U/μl *Taq* DNA polymerase (Takara) (final concentration), and 1 μl of bacterial suspension or cDNA. PCR was conducted using a MyCycler thermocycler (Life Science). The PCR program for primers pbpA‐F/pbpA‐R, gapA‐F/gapA‐R, bipA‐F/bipA‐R, tufA‐F/tufA‐R, rpoB‐F/rpoB‐R, and chpC‐F/chpC‐R was as follows: 95ºC for 10 min, followed by 35 cycles of 95°C for 60 s, 58‐62°C for 60 s and 72°C for 90–240 s, and a final extension of 10 min at 72°C (the annealing temperatures and extension times were distinct for different primer sets, Table A1 in Appendix), while the gyrB5754F/gyrB8082R and the celA17558F/celA19998R assays were conducted as previously described (Luo, 2008). A 516‐bp amplicon was obtained for the primer pair rRNA‐I/rRNA‐O using the following thermal profile: 95°C for 5 min, followed by 35 cycles of 95°C for 40 s, 54°C for 40 s and 72°C for 40 s, and a final extension of 10 min at 72°C. The resulting products were visualized with ultraviolet illumination after electrophoresis in a 1% (w/v) agarose gel and stained in 1 µg/ml of ethidium bromide (EB) solution for 10 min.

The 20 µl quantitative PCR reaction mixtures contained 10 μl of 2 × SYBR Premix Ex *Taq* Ⅱ (Takara), 0.4 μl 50× ROX Reference Dye (Takara), 2 μl cDNA template, and varying final concentration of each primer (0.5 μM of gyrBRT‐F/gyrBRT‐R, pbpART‐F/pbpART‐R and bipART‐F/bipART‐R or 0.4 μM of gapART‐F/gapART‐R, tufART‐F/tufART‐R, rpoBRT‐F/rpoBRT‐R, chpCRT‐FR/chpCRT‐R and celART‐F/celART‐R). The qPCR was performed using a 7500 Real‐time PCR system (Thermo Fisher Scientific) and the thermal conditions were 95°C for 60 s, followed by 40 cycles of 95ºC for 10 s, 55°C for 30 s, and 72°C for 30 s. To analyze the specificity of the primers, melt curve analyses were conducted immediately after the 40 PCR cycles using the default thermal profile (95°C for 15 s, 60°C for 60 s, then slowly increasing the temperature to 95°C at a 1% ramp rate with continuous measurement of fluorescence, and a final incubation at 60°C for 15 s). All experiments were done in triplicate, and three nontemplate controls were included for each primer pair.

### 2.5 Data analysis and normalization of pathogenicity genes

Software geNorm and NormFinder were used to evaluate the expression stability of 6 reference gene candidates. geNorm estimates the most stably expressed reference genes through a stepwise exclusion or ranking process, and the normalization factor is the geometric mean of the most stable reference genes (Vandesompele et al., 2002). NormFinder is an add‐in tool for Excel, which considers the intragroup and intergroup variations of the sample sets. It will show the best reference gene or the best combination of two genes regards to expression stability (Andersen et al., 2004). The cycle threshold (*C*_t_) value generated by real‐time PCR should be transformed before analysis by geNorm and NormFinder. geNorm calculated the gene expression stability measure (M), which is the mean pairwise variation for a gene from all other control genes, and the pairwise variation (*V*_n/n+1_) (Vandesompele et al., 2002). Stably expressed genes display low M values. The proposed threshold for choosing stable genes was M ≤ 0.7 and V ≤ 0.15 (Nguewa et al., 2008; Vandesompele et al., 2002). NormFinder calculated the gene expression stability value (SV) and considered both the intragroup variation and the intergroup variation. The gene with the most stable expression has the lowest SV. The normalization factor (NF) was calculated as the geometric mean of the Ct value of selected reference genes (Vandesompele et al., 2002).

## 3 RESULTS

### 3.1 Selection of reference gene candidates and their qRT‐PCR primers

The reference gene candidates used in this study were selected based on two criteria, (a) genes routinely used in bacteria for transcript normalization and (b) genes described as stably expressed under environmental stress. The housekeeping genes including *gyrB* (DNA gyrase B subunit), *pbpA* (penicillin‐binding protein 2), *gapA* (glyceraldehyde‐3‐phosphate dehydrogenase), *bipA* (membrane GTPase), *tufA* (translation elongation factor Tu), and *rpoB* (DNA‐directed RNA polymerase beta subunit) were chosen for this study. These genes had different functions to avoid problems associated with coregulation. The *celA* (cellulase) and *chpC* (serine protease) genes*,* which are important for *C*. *michiganensis* pathogenicity, were selected as validating genes and used to evaluate the applicability of reference genes.

Six primer pairs that amplified *pbpA*, *gapA*, *bipA*, *tufA*, *rpoB,* and *chpC* genes (Table A1 in Appendix) were designed according to the genome sequence of *C*. *michiganensis* strain NCPPB382. The sequences of 6 reference gene candidates and 2 pathogenicity genes of BT0505 were aligned to the genome sequence of strain NCPPB382 using Basic Local Alignment Search Tool (BLAST) on National Center for Biotechnology Information (NCBI). The complete sequences of eight selected genes of *C*. *michiganensis* strain BT0505 were deposited in GenBank, and the accession numbers were listed in Table 1. These sequences were used for designing primers for qPCR. The variation of these eight genes between BT0505 and NCPPB382 was listed in Table A2 in Appendix.

**Figure A1:**
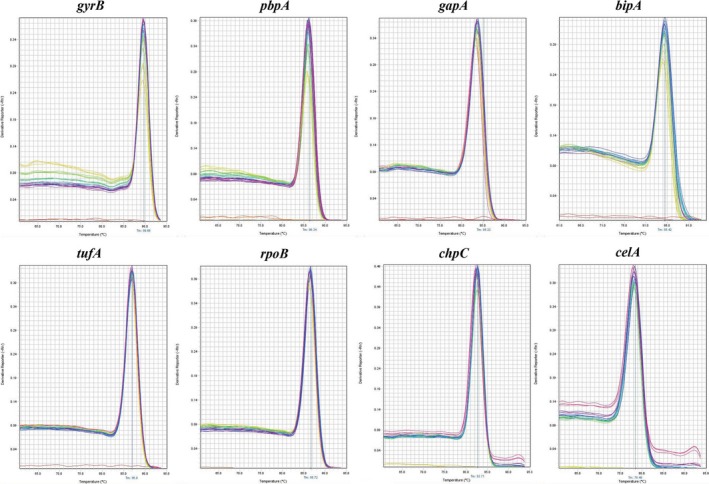
Melt curve analysis of six reference gene candidates and two pathogenicity genes of *Clavibacter michiganensis*

**Figure A2:**
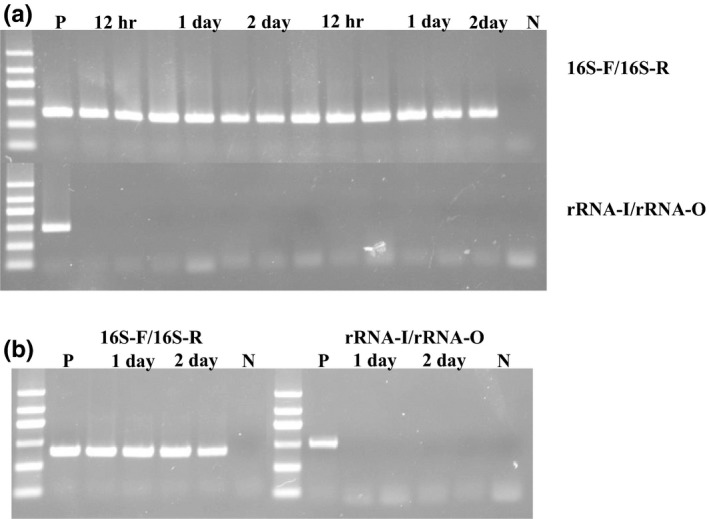
PCR test result of DNA. Total RNA was reverse transcript to cDNA and test by specific PCR primers. 16S‐F/R targeted to 16S rRNA while rRNA‐I/rRNA‐O targeted to the space between rRNA and *tyrS* (tyrosyl‐tRNA synthetase) sequences which were not in the same transcript. (a) Samples treated with 0.85% NaCl. (b) Samples treated with CuSO_4_. P, genomic DNA of *Clavibacter michiganensis*. N, negative control

Real‐time qRT‐PCR was optimized for each primer pair (Table 1) using cDNA samples. The primer pair rRNA‐I/rRNA‐O designed in this study was used to confirm the absence of gDNA. The 516‐bp amplicon included the space between rRNA and *tyrS* (tyrosyl‐tRNA synthetase) sequences which was not in the same transcript. All cDNA samples were negative for the 516‐bp amplicon, indicating that the cDNA samples were free of gDNA (Figure A2 in Appendix). Melt curve analyses showed a single melting peak for each primer pair in qPCR with the *C*. *michiganensis* cDNA (Figure A1 in Appendix). This indicated that there was no nonspecific amplification with the primer pairs. Amplification efficiency values ranged from 91% to 101% (Table 1) confirmed that these 6 primer pairs were suitable for evaluating the expression stability of the reference gene candidates.

### 3.2 Expression stability of the reference gene candidates in cultural conditions

**Figure 1:**
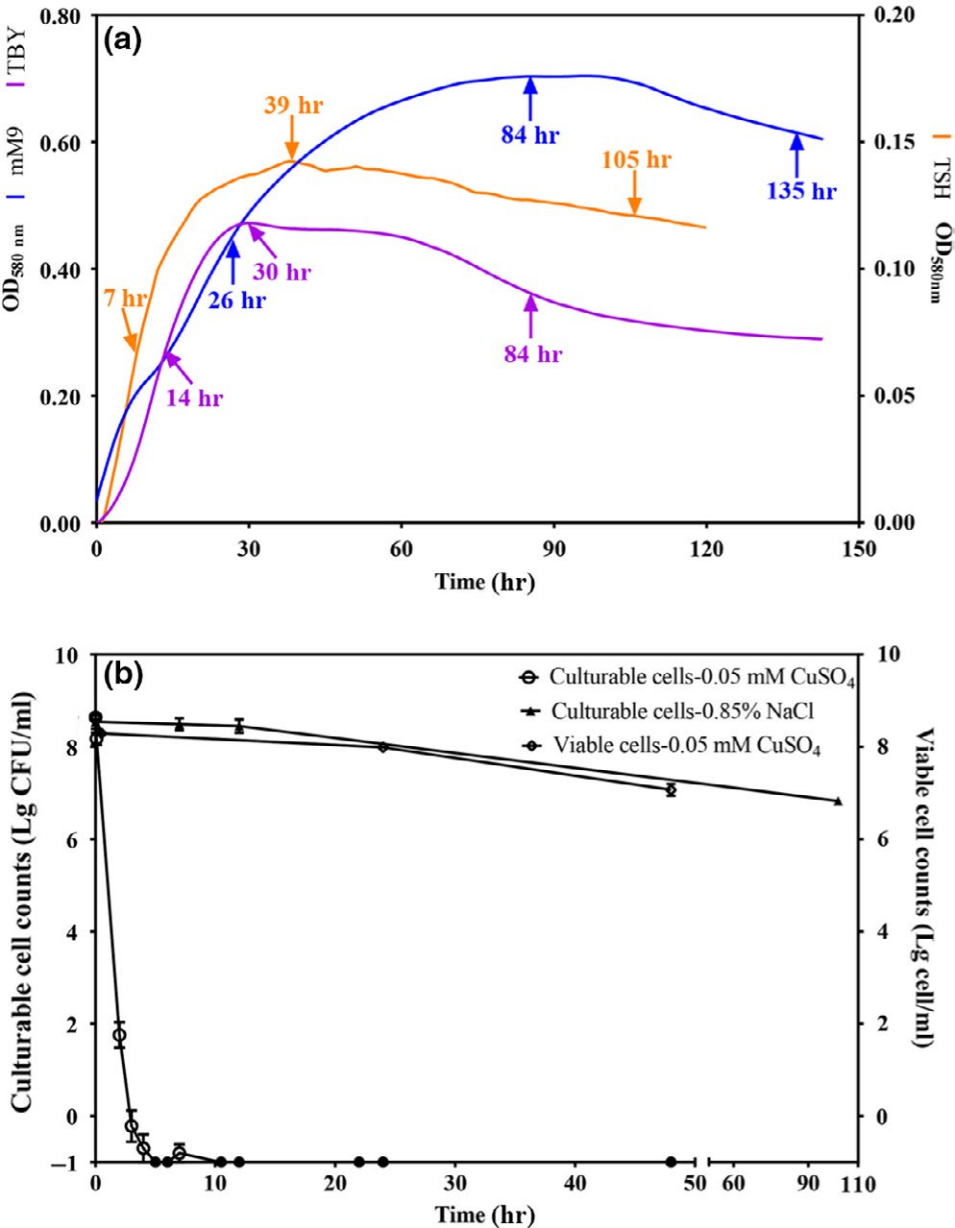
Population growth of *Clavibacter michiganensis* in five experimental conditions. (a) Three cultural conditions, TBY broth (purple), modified M9Cmm‐minimal medium (mM9, blue), and tomato seedling homogenate (TSH, orange). The curves were generated using a BioScreen C Pro Automated Microbiology Growth Curve Analysis System. Time points on each curve represented the log phase, stationary phase, and decline phase from left to right. (b) Survival curve of *Clavibacter michiganensis* in 0.85% NaCl solution (triangle) and 0.85% NaCl solution supplemented with 50 μM CuSO_4_ (circle). Culturable cell counts were determined by the plating method, and viable cells counts were calculated by flow cytometry method. Error bars represent the standard deviation of two biological replicates

**Figure 2:**
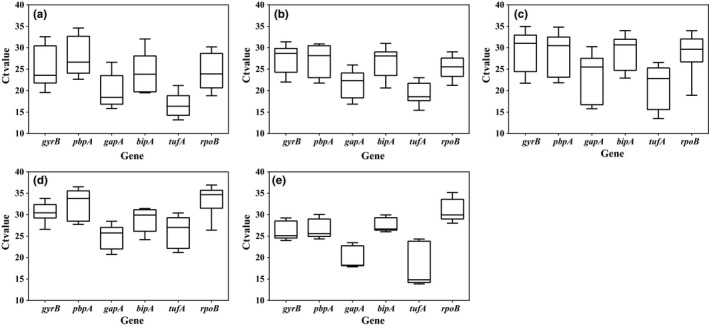
Expression profiles of *Clavibacter michiganensis* reference gene candidates. Expression data are displayed as *C*_t_ values for each reference gene in different experimental conditions. The line across the box represents the median. The box indicates the 25th and 75th percentiles of *C*_t_ values. Whiskers represent the 1th and 99th percentiles of Ct values. (a) TBY broth. (b) mM9 medium. (c) TSH medium. (d) 0.85% NaCl solution. (e) 0.85% NaCl solution supplemented with 50 μM CuSO_4_

**Figure 3:**
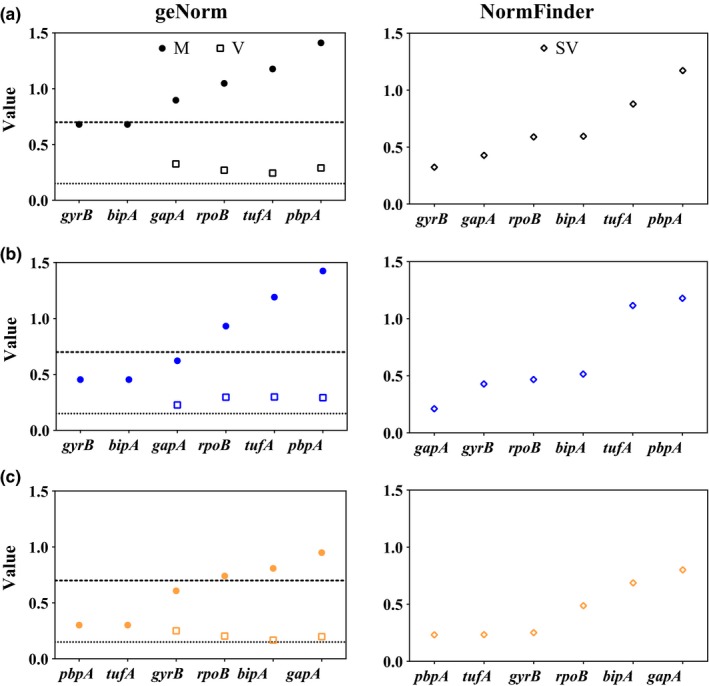
Expression stability of *Clavibacter michiganensis* reference gene candidates in culturable samples ranked by geNorm (left) and NormFinder (right). (a) TBY broth. (b) mM9 medium. (c) TSH medium. M, stability measure. V, pairwise variation. and SV, stability value. The upper and lower dash lines in left figures represent the cut‐off M value (0.7) and V value (0.15), respectively

The expression stability of six candidates of *C*. *michiganensis* was evaluated in three different experimental conditions at the log, stationary, and decline growth phases. *C*. *michiganensis* grew fastest in TSH medium, followed by TBY broth and mM9 medium at the log phase. However, the greatest biomass was obtained in mM9 medium at the stationary phase (Figure 1a). The expression levels of the six candidate genes were presented as the raw *C*_t_ values. The ranges of expression were 19.59–34.97, 21.77–34.82, 15.78–30.24, 19.50–34.00, 13.19–26.57, and 18.82–33.97 for *gyrB*, *pbpA*, *gapA*, *bipA*, *tufA,* and *rpoB*, respectively (Figure 2a‐c). The *gyrB* and *bipA* got the lowest M value 0.680 followed by *gapA* (0.897) and *rpoB* (1.048), and the combination of these 4 genes yielded the lowest V value (0.244). *tufA* and *pbpA* were ranked as the most unstable genes by both geNorm and NormFinder software packages (Figure 3a). According to the cut‐off *M* ≤ 0.7, *gyrB* and *bipA* were suitable reference genes, even though *bipA* was ranked 4th according to NormFinder.

In TBY broth, the average Ct values were 25.26 ± 4.56, 27.96 ± 4.38, 19.89 ± 3.87, 24.34 ± 4.60, 16.81 ± 2.81, and 24.06 ± 3.84 for the expression of *gyrB*, *pbpA*, *gapA*, *bipA*, *tufA,* and *rpoB*, respectively in *C*. *michiganensis* (Figure 2a). In mM9 medium, however, the *C*_t_ values were 27.44 ± 3.04, 27.10 ± 3.56, 21.72 ± 3.08, 26.65 ± 3.34, 19.22 ± 2.36, and 25.20 ± 2.52 for the expression of *gyrB*, *pbpA*, *gapA*, *bipA*, *tufA,* and *rpoB*, respectively (Figure 2b). The results show that these genes displayed lower expression levels, but greater expression stability in mM9 medium although the differences were not significant according to *t* test (*p* < .05). In geNorm analyses, *gyrB* (0.454), *bipA* (0.454), and *gapA* (0.622) fit the cut‐off limit of *M* ≤ 0.7, but the lowest *V* value was 0.227 (*V*_2/3_), which was greater than the threshold for *V* (≤0.15) (Figure 3b). The ranking of the genes based on expression stability was different between NormFinder and geNorm. According to NormFinder, the most stably expressed gene was *gapA* and the least stably gene was *pbpA* (Figure 3b). Based on geNorm, *gyrB*, *bipA,* and *gapA* were the most suitable reference gene candidates despite the fourth ranking of *bipA* by NormFinder.

The expression stability of *C*. *michiganensis* reference genes in TSH medium was different to that in TBY broth and mM9 medium. The average Ct values were 29.35 ± 4.62, 28.73 ± 4.57, 23.34 ± 5.29, 19.12 ± 3.90, 21.13 ± 4.74, and 28.52 ± 4.79 for the expression of *gyrB*, *pbpA*, *gapA*, *bipA*, *tufA,* and *rpoB* in TSH medium, respectively (Figure 2c). Interestingly, *pbpA,* which was the least stably expressed gene in TBY and mM9 media, was the most stably expressed gene in TSH medium (Figure 3c). Analyses by both geNorm and NormFinder showed the same rankings for expression stability of the candidate reference genes. *pbpA* and *gapA* were the most and least stably expressed genes, respectively.

The top three stably expressed genes, *pbpA*, *tufA,* and *gyrB*, were suitable for transcript normalization in vitro according to the criteria described above. Combined all results, the *gyrB* was an optimal reference gene for culture‐based research. Moreover, the *bipA* and *gapA* were also recommended for in vitro research, such as incubation in TBY broth and mM9 medium. However, *pbpA*, *tufA,* and *gyrB* were an appropriate reference gene set in *C*. *michiganensis*—tomato interaction research (Table 2).

**Table 2:** Recommended reference genes for in vitro research of *Clavibacter michiganensis*

Experimental condition	Recommended reference genes				
In vitro culture	*gyrB*	*bipA*	*gapA*		
Interaction with tomato	*pbpA*	*tufA*	*gyrB*		
Starvation	***bipA***	***rpoB***	*gapA*	*pbpA*	*tufA*
VBNC	***gyrB***	***pbpA***	***rpoB***	*gapA*	*bipA*

Note

The names of genes in bold represent the best combination of reference genes.

### 3.3 Expression stability of the reference gene candidates in oligotrophic conditions

The expression stability of six *C*. *michiganensis* reference genes was evaluated in 0.85% NaCl solution and 0.85% NaCl solution supplemented with 50 μM CuSO_4_, which represent starvation and VBNC‐inducing conditions, respectively. In 0.85% NaCl solution, the population of culturable cells decreased slightly, from 10^8^ CFU/ml to 10^7^ CFU/ml within 4 days (Figure 1b). Under the presence of copper ions, however, the culturable cells decreased below the limit of detection (0.1 CFU/ml) at 12 hr postinduction, and the viable cells remained constantly at 10^7^ cell/ml by 2 d after induction, suggesting that the cells were in VBNC state (Figure 1b).

For the two oligotrophic environments, the Ct values of the six candidate genes were 23.97–33.80, 24.36–36.51, 17.85–28.47, 24.20–31.46, 13.86–30.42, and 26.40–36.92 for *gyrB*, *pbpA*, *gapA*, *bipA*, *tufA,* and *rpoB*, respectively. Compared to the 0.85% NaCl solution, the standard deviations of *C*_t_ values for expression of all genes except *rpoB* were smaller in the copper‐stress environment (Figure 2d,e). Based on the geNorm analyses, the *gyrB* and *tufA* genes did not meet the threshold for acceptance (*M* ≤ 0.7) in 0.85% NaCl solution and copper‐stress conditions, respectively (Table 3). The *V*_2/3_ value for *bipA* and *rpoB* (*V*_2/3_ = 0.09) and the *V*_3/4_ value of *gyrB*, *pbpA,* and *rpoB* (*V*_3/4_ = 0.14) were less than 0.15 in 0.85% NaCl solution and the copper‐stress condition, respectively (Table 3). The combination of *bipA* and *rpoB* and the combination of *gyrB*, *pbpA,* and *rpoB* were sufficiently stable to serve as normalization factors for measuring *C*. *michiganensis* gene expression in starvation and VBNC studies, respectively. NormFinder ranked *rpoB* and *gyrB* as the most stably and most unstably expressed genes in 0.85% NaCl solution, respectively. In the copper‐stress condition, geNorm and NormFinder were in agreement with regards to the rank of six genes (Table 3). These results indicated *bipA* and *rpoB* were consistently found to be the most suitable reference genes for *C*. *michiganensis* gene expression studies in starvation conditions, and the combination of *gyrB*, *pbpA,* and *rpoB* was recommended for VBNC research (Table 2).

**Table 3:** Expression stability of *Clavibacter michiganensis* reference gene candidates in oligotrophic samples

Nutrient starvation^a^			VBNC^b^			Nutrient starvation		VBNC	
Gene	M	V	Gene	M	V	Gene	SV	Gene	SV
*bipA*			*gyrB*			*rpoB*	0.06	*gyrB*	0.10
*rpoB*	0.16	0.09	*pbpA*	0.11	0.18	*gapA*	0.09	*pbpA*	0.14
*gapA*	0.24	0.25	*rpoB*	0.39	0.14	*bipA*	0.26	*rpoB*	0.17
*pbpA*	0.62	0.15	*gapA*	0.50	0.17	*tufA*	0.60	*gapA*	0.18
*tufA*	0.70	0.26	*bipA*	0.65	0.52	*pbpA*	0.63	*bipA*	0.88
*gyrB*	1.01		*tufA*	1.47		*gyrB*	1.08	*tufA*	2.15

Note

Stability measure (M) and pairwise variation (V) were calculated with geNorm. Stability value (SV) was estimated with NormFinder.

a 0.85% NaCl solution.

b 0.85% NaCl solution supplemented with 50 μM CuSO_4_.

### 3.4 Validation of reference genes

**Figure 4:**
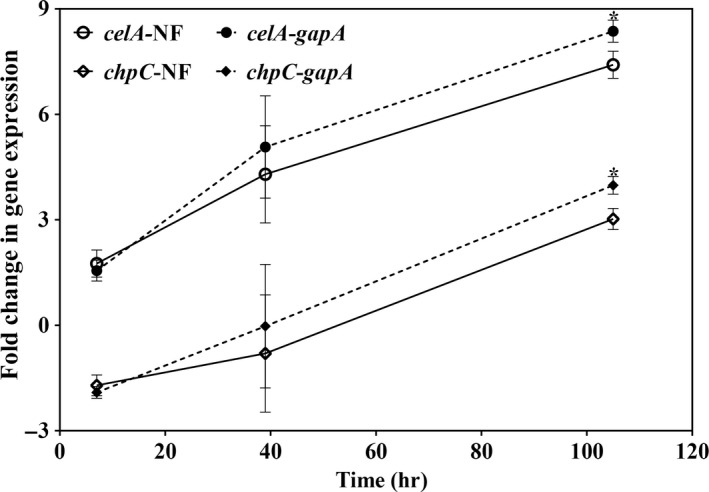
Relative expression levels of two *Clavibacter michiganensis* pathogenicity genes in TSH medium. The sample collected at the log phase in TBY broth was used as control. The normalization factor (NF) was calculated as the geometric mean of *C*_t_ values of three most stable genes (*pbpA*, *tufA,* and *gyrB*) in tomato seedling homogenate. The relative expression levels of *celA* and *chpC* were normalized to NF (solid line, blank symbol) and the least stable gene *gapA* (dash line, dark symbol), respectively. The fold change in gene expression was calculated as—ΔΔ*C*_t_. Error bars show standard deviation calculated from three biological replicates. Asterisks represent significant differences between the relative expression levels of pathogenicity genes normalized to NF and *gapA* according to a two‐sided one sample *t* test (*p* < .05)

In order to validate the selected reference genes, the relative expression level of two *C*. *michiganensis* pathogenicity genes, *celA,* and *chpC*, were evaluated in TSH medium. *Clavibacter* *michiganensis* cells collected at the log phase in TBY broth were used as control. In TSH medium, the expression of *celA* and *chpC* were normalized using the three most stable reference genes (*pbpA*, *tufA,* and *gyrB*) and the least stable gene (*gapA*), respectively. *celA* expression increased over time. In contrast, *chpC* expression was down‐regulated initially then increased. Normalized of gene expression with the most suitable reference genes (*pbpA*, *tufA,* and *gyrB*) or *gapA*, revealed that expression of both *celA* and *chpC* increased over time in TSH medium (Figure 4). However, comparing with the three most suitable reference genes, the expression of *chpC* and *celA* are higher when only *gapA* gene was used for normalization. Moreover, the difference at 105 hr was significant according to *t* test (*p* < .05). These results indicate that unsuitable reference genes may lead to an overestimation of pathogenicity gene expression in *C*. *michiganensis*.

## 4 DISCUSSION

Real‐time qRT‐PCR, a simple method for quantifying gene expression, plays an important role in molecular biology. However, accurate quantification of gene expression should be based on the stable expression of reference genes (Zhou et al., 2011). Unfortunately, it is difficult to find a universal reference gene stably expressed under different environmental conditions for bacteria. Bacterial gene expression varies widely depending on the experimental treatments (Takle et al., 2007). Hence, it is critical to screen appropriate reference genes under the experimental condition to confirm stable gene expression. Here, we empirically assessed the expression stability of six reference genes in *C*. *michiganensis* under five experimental conditions.

Six housekeeping genes, *gyrB*, *pbpA*, *gapA*, *bipA*, *tufA,* and *rpoB*, were evaluated in this study. The *gyrB* gene, encoding DNA gyrase, is a common reference gene especially for Gram‐positive bacteria and is stably expressed in nutrient‐rich media or under certain stress conditions (Carvalho et al., 2014; Crawford, Singh, Metcalf, Gibson, & Weese, 2014; Duquenne et al., 2010; Sihto, Tasara, Stephan, & Johler, 2014). The *gapA* gene, encoding glyceraldehyde‐3‐phosphate dehydrogenase, is a key gene for metabolism and has been used to normalize the expression of virulence genes (Bjelland et al., 2013; Kjeldgaard, Henriksen, Cohn, Aabo, & Ingmer, 2011). We observed that M value of g*yrB* and *gapA* was 0.82 (data not shown), which suggests that these genes are suitable for some studies (Botteldoorn et al., 2006; Mafra et al., 2012). These two genes can be used as reference genes for *C. michiganensis* when the bacteria are cultured under most common conditions, such as TBY broth and other nutrient‐rich media.

The *pbpA* gene, which is responsible for synthesizing the penicillin‐binding protein, expressed stably under different conditions, including interaction with host cells, nutrient starvation, and VBNC induction. Previously, *pbp5*, which is a homolog of *pbpA*, was successfully used as a reference gene for studying the viability of VBNC *Enterococcus faecalis* cells (Lleò et al., 2001). *bipA* and *gyrA* were reported as reference genes in *C. michiganensis*—host interaction study (Chalupowicz et al., 2017, 2010). In current study, we showed that *pbpA* and *tufA* were better reference genes than *bipA* in TSH medium, which is a mimic of natural host‐pathogen interaction condition (Figure 3c). Interestingly, *tufA* and *rpoS* genes were constitutively expressed in VBNC *Vibrio vulnificus* cells (Smith & Oliver, 2006). *rpoB* is a homolog gene of *rpoS* in *C. michiganensis*, and its M value was less than 0.5 under nutrient starvation and VBNC‐inducing conditions, indicating that *rpoB* is a suitable reference gene under stressful conditions. On the other hand, *tufA* expression was less stable than *rpoB* when *C*. *michiganensis* was grown under stress conditions.

Furthermore, the commonly used reference gene, *16S rRNA*, was ruled out in our study. Since rRNA accounts for more than 95% of bacterial total RNA, and the expression level of rRNA is significantly higher than that of mRNA (Peano et al., 2013). Additionally, rRNA is more stable than mRNA, and its quantity is not comparable to that of mRNA (Deutscher, 2006; Peano et al., 2013). The use of rRNA as reference gene for a human tissue biopsy was also found to be inappropriate (Tricarico et al., 2002).

The expression of *C*. *michiganensis* reference genes tested in this study varied among the experimental conditions, confirming that standard housekeeping genes should be screened before use for normalization of gene expression. Based on *t* test (*p* < .05), we observed significant difference when normalizing the expression of *celA* and *chpC* with different normalization factors, the three most stable reference genes (*pbpA*, *tufA,* and *gyrB*) and the most variable gene (*gapA*) at 105 hr in TSH medium (Figure 4). Similar results were reported in studies using reference genes of melon and citrus (Kong et al., 2014; Mafra et al., 2012). The use of inappropriate reference genes led to an increasing fold change of *WRKY70*, which was upregulated 35‐fold compared to the most stable reference genes (Mafra et al., 2012). These results indicate that inappropriate reference genes can lead to a significant misinterpretation of data.

geNorm and NormFinder ranked the six *C*. *michiganensis* reference genes differently in all experimental conditions but the TSH medium and VBNC state. Other studies also reported that the use of different software could lead to different rankings of reference genes (Cruz et al., 2009; Kong et al., 2014; Mafra et al., 2012). These differences may be caused by the use of different statistical algorithms (Andersen et al., 2004). NormFinder ranked the most stable genes with minimal inter‐ and intra‐group variation and geNorm selected the top two genes with the highest similarity in expression and the lowest intragroup variation (Andersen et al., 2004; Vandesompele et al., 2002). Thus, coregulated genes should be excluded when using geNorm to screen reference genes.

## 5 CONCLUSION

In summary, we systematically screened *C. michiganensis* reference genes for use in real‐time qRT‐PCR. The *gyrB* and *gapA* genes stably expressed in most of the experimental conditions except in 0.85% NaCl solution and TSH medium. This highlights the need to empirically assess the suitability of reference genes under different experimental conditions before use. We found that *gyrB*, *bipA,* and *gapA* were suitable for artificial nutrient conditions. On the other hand, *pbpA*, *tufA,* and *gyrB* were suitable for use as reference genes in pathogen‐host interaction studies. The combination of *bipA* and *rpoB* and the combination of *gyrB*, *pbpA,* and *rpoB* would be useful for nutrient starvation and VBNC experiments, respectively. Because of the similarity of *C. michiganensis* and other phytopathogenic bacteria, this study provides useful information on the reference genes selection for other researches, especially for studies related with plant pathology.

## CONFLICT OF INTERESTS

The authors declare no conflict of interests.

## AUTHOR CONTRIBUTIONS

Conceptualization: Laixin Luo, Ronald Walcott; Data Curation: Na Jiang, Qingyang Lyu; Funding Acquisition: Laixin Luo, Jianqiang Li; Investigation: Na Jiang, Qingyang Lyu, Xin Xu; Methodology: Na Jiang, Qingyang Lyu, Sining Han; Project Administration: Laixin Luo; Supervision: Laixin Luo, Jianqiang Li; Writing‐Original Draft Preparation: Na Jiang; Writing‐review & editing: Qingyang Lyu, Ronald Walcott.

## ETHICAL APPROVAL

None required.

## Data Availability

All data are included in the main manuscript and in the appendices. Raw data and materials are available on request.

## APPENDIX 1

**Table A1:** Primers for copying reference and target genes of *Clavibacter michiganensis* strain BT0505

Gene name	Primer ID	Primer sequence (5′→3′)	Annealing temperature (ºC)	Extension time (s)	Amplicon size (bp)
*gyrB*	gyrB5754F	ATACCTACGGCTAGGGAGG	57	150	2,329
	gyrB8082R	AGGTCCCGTTCAGAGGTC			
*pbpA*	pbpA‐F	TCCTCCTCGCCGTAGTC	60	120	1,964
	pbpA‐R	ACCTGCTGCTCGTCTCG			
*gapA*	gapA‐F	GGGACGCAGGCTGTACTT	62	90	1,297
	gapA‐R	CCGAACCTCCATCACCC			
*bipA*	bipA‐F	ACCTTGCCGAGCGTGT	58	130	1,261
	bipA‐R	TTCGCCCTCCTCCCTT			
*tufA*	tufA‐F	GCACCATCGCCATCGGT	59	90	1,425
	tufA‐R	GCATCCGCCGTTTCAGT			
*rpoB*	rpoB‐F	TCAGCGTGCGGTAGTTG	60	240	3,806
	rpoB‐R	GGTCGGTCATCGTGTGC			
*chpC*	chpC‐F	CGAGACGGCTGCCCGCAAACTA	61	90	1,248
	chpC‐R	CCGCGAGACGAGCGTGGAAACA			
*celA*	celA17558F	GAGGTCCTCGGCTCTGAG	58	150	2,441
	celA19998R	CTGCATGACGAGAGATGTC			

Note

The gyrB5754F/gyrB8082R primers and the celA17558F/celA19998R primers were described by Luo (2008) and the other six primer pairs were designed in this study.

**Table A2:** Variation of genes used in this study from BT0505 compared to NCPPB382

Gene	DNA difference	Amino acid difference	G + C (%)	GenBank accession number
*gyrB*	5/2046	3/681	67.2	KJ123741
*pbpA*	2/1452	0/483	69.4	KJ123735
*gapA*	2/1008	0/335	68.0	KJ123733
*bipA*	2/1908	0/635	68.3	KF990998
*tufA*	0/1194	0/397	66.6	KJ123740
*rpoB*	4/3489	0/1162	67.3	KJ123738
*chpC*	1/861	0/286	52.0	KJ123731
*celA*	9/2241	3/746	66.8	KJ123730
